# Supplementary material for: Unspecific Molecular Adsorption (UMA) Sample Preparation Method for Bottom‐up and Whole Protein Analysis: The Foundation
Source: Proteomics. 2026 May 26;26(10):6–15. doi: 10.1002/pmic.70149 (PMC13615483; doi:10.1002/pmic.70149)
Supplement: Supplementary file 2 [file PMIC-26--s002.zip › pmic70149-sup-0001-Figures_Methods_Protocols/Supplementary_protocols_UMA_0505026_.docx]

**Supplementary Protocol 1**

Basic UMA Tip Digestion Protocol

1) Cells are lysed in the basic SDS solubilization buffer (5% sodium dodecyl sulphate, 50 mM triethylammonium bicarbonate, pH ~8.0).

2) DNA is sheared by probe sonication. The sample is clarified by centrifugation for 5 min at ~13,000xg.

3) Protein disulphide bonds are reduced and cysteine residues alkylated (e.g. incubation with 20 mM dithiothreitol at 50 °C for 30 min and consequent incubation for 30 min at room temperature in the dark with 40 mM iodoacetamide).

4) The UMA tip can handle up to ~50 µg of protein load.

5) Add 6 volumes of the AMA solution (45% acetonitrile, 45% methanol, 100 mM ammonium acetate) to the sample. Mix by pipetting up and down several times. Add the mix into the UMA tip.

6) Spin the UMA tip in a bench-top centrifuge in a 2.0 ml sample tube with a tip holder at ~2,500xg until all solution has passed through.

7) Wash by adding 150 µL of the AMA solution to the UMA tip. Spin at 5,000xg until all solution has passed through. The optional additional wash with the AMA solution followed by the wash with the 70 mM ammonium bicarbonate could be performed after decanting the flow-through waste from the 2-ml tube into an appropriate waste container.

8) Add 2 µg of trypsin in 40 µl of 70 mM ammonium bicarbonate into the UMA tip. Place the UMA tip into a 1.5 ml sample collection tube. Push about half of the trypsin solution through the UMA tip using a syringe with a tip adapter. Incubate the UMA tip/tube at 53 °C for 45 min.

9) Elute peptide products with 40 µL of 70 mM ammonium bicarbonate.

10) Optional. Additional elution with 40 µL of 30% acetonitrile in 0.5% formic acid. Combine with the first elution.

11) Dry down the obtained eluate and re-suspend in 1% acetonitrile, 0.2% formic acid.

**Supplementary Protocol 2**

Basic UMA-P (UMA-Pipetting) Digestion Protocol

1) Cells are lysed in the basic SDS solubilization buffer (5% sodium dodecyl sulphate, 50 mM triethylammoniumcarbonate, pH ~8.0).

2) DNA is sheared by probe sonication. The sample is clarified by centrifugation for 5 min at ~13,000xg.

3) Protein disulphide bonds are reduced and cysteine residues alkylated (e.g. incubation with 20 mM dithiothreitol at 50 °C for 30 min and consequent incubation for 30 min at room temperature in the dark with 40 mM iodoacetamide).

4) The UMA-P tip can handle up to ~50 µg of protein load.

5) Add 5 volumes of the AMA solution (45% acetonitrile, 45% methanol, 100 mM ammonium acetate) to the sample.

6) Pipet the mixture through the UMA-P tip up and down 30 times. Discard the mixture into an appropriate waste container.

7) Wash by pipetting and discarding 100 µl of the AMA solution through the UMA-P tip three times. Dispose the used AMA solution into an appropriate waste container.

8) Add 2 µg of trypsin in 30 µl of 70 mM ammonium bicarbonate into a 1.5 ml sample tube. Place the UMA-P tip into this trypsin solution and draw the solution into the tip. Incubate the UMA-P tip/tube at 53 °C for 30 min.

9) Elute peptides by pipetting the digestion solution out of the UMA-P tip.

10) Dry down the obtained eluate and re-suspend in 1% acetonitrile, 0.2% formic acid.

**Supplementary Protocol 3**

Basic UMA-R Tip Protein Recovery Protocol

The UMA-R tip is used for protein clean-up, capture and elution onto a wide-pore reversed phase

1) Cells are lysed in the basic SDS solubilization buffer (5% sodium dodecyl sulphate, 50 mM triethylammonium bicarbonate, pH ~8.0).

2) DNA is sheared by probe sonication. Sample is clarified by centrifugation for 5 min at ~13,000xg.

3) Optional step. Protein disulphide bonds are reduced and cysteine residues alkylated (e.g. incubation with 20 mM dithiothreitol at 50°C for 30 min and consequent incubation for 30 min at room temperature in the dark with 40 mM iodoacetamide).

4) The UMA-R tip can handle up to ~50 µg of protein load.

5) Add 6 volumes of the AMA solution (45% acetonitrile, 45% methanol, 100 mM ammonium acetate) to the sample. Mix by pipetting up and down several times. Add the mix into the UMA-R tip.

6) Spin the UMA-R tip in a bench-top centrifuge in a 2.0 ml sample tube with a column holder at ~2,500xg until all solution has passed through.

7) Wash by adding 150 µL of the AMA solution to the UMA-R tip. Spin at 3,000xg until all solution has passed through. Discard the flow-through into an appropriate waste container.

8) The captured proteins are forwarded onto the reversed phase part of the UMA-R tip:

Option 1 (preferred). EA protocol. Add 150 µL of 100 mM ammonium acetate. Spin at 3,000xg until all solution has passed through. Add 150 µL of 20% ethanolamine (EA). Wait 2 min (optional incubation at 70°C may improve protein recovery). Spin at 2,000xg until all solution has passed through. Add 150 µL of 3% acetonitrile. Spin at 3,000xg until all solution has passed through. Discard the flow-through into a designated waste container. Ethanolamine is classified as a hazardous substance and should be dealt with accordingly.

Option 2. TT protocol. Add 150 µL of 100 mM ammonium acetate. Spin at 3,000xg until all solution has passed through. Add 150 µL of 70% trifluoroethanol in 100 mM triethylammonium bicarbonate. Wait 2 min. Spin at 2,000xg until all solution has passed through. Add 150 µL of 3% acetonitrile. Spin at 3,000xg until all solution has passed through. Discard the flow-through into a designated waste container. Trifluoroethanol is classified as a hazardous substance and should be dealt with accordingly.

Option 3. FA protocol. Add 150 µL of 0.5% formic acid. Spin at 3,000xg until all solution has passed through. Add 150 µL of 50% formic acid. Wait 2 min. Spin at 2,000xg until all solution has passed through. Add 150 µL of 3% acetonitrile. Spin at 3,000xg until all solution has passed through. Discard the flow-through into an appropriate waste container. Formic Acid is classified as a hazardous substance and should be dealt with accordingly.

9) The proteins captured on the reversed phase of the UMA-R tip could be eluted with increased acetonitrile or acetonitrile/alcohol mixture concentrations in 0.1% trifluoroacetic acid.

**Supplementary Protocol 4**

Basic UMA Tip Protein Capture and Elution protocol

1) Proteins are solubilized in the basic SDS solubilization buffer (5% sodium dodecyl sulphate, 50 mM triethylammonium bicarbonate, pH ~8.0).

2) Optional step. Protein disulphide bonds are reduced and cysteine residues alkylated (e.g. incubation with 20 mM dithiothreitol at 50 °C for 30 min and consequent incubation for 30 min at room temperature in the dark with 40 mM iodoacetamide).

3) The UMA tip can handle up to ~50 µg of protein load.

4) Add 6 volumes of the AMA solution (45% acetonitrile, 45% methanol, 100 mM ammonium acetate) to the sample. Mix by pipetting up and down several times. Add the mix into the column.

5) Spin the UMA tip in a bench-top centrifuge in a 2.0 ml sample tube with a tip holder at ~2,500xg until all solution has passed through.

6) Wash by adding 150 µL of the AMA solution to the UMA tip. Spin at 5,000xg until all solution has passed through.

7) Elute proteins with 30 µL of either 0.1% trifluoroacetic acid or 35% trifluoroethanol in 1% formic acid or 70% trifluoroethanol in 1% formic acid solution (1% formic acid could be substituted with 0.1% trifluoroacetic acid). An optional incubation in the elution solution for 2 min at RT could improve protein recovery.

Important Note: Trifluoroethanol is classified as a hazardous substance and should be dealt with accordingly.

**Supplementary Protocol 5**

Basic UMA-P Tip Pipetting Protein Capture and Elution protocol

1) Proteins are solubilized in the basic SDS solubilization buffer (5% sodium dodecyl sulphate, 50 mM triethylammonium bicarbonate, pH ~8.0).

2) Optional step. Protein disulphide bonds are reduced and cysteine residues alkylated (e.g. incubation with 20 mM dithiothreitol at 50 °C for 30 min and consequent incubation for 30 min at room temperature in the dark with 40 mM iodoacetamide).

3) The UMA-P tip can handle up to ~50 µg of protein load.

4) Add 5 volumes of the AMA solution (45% acetonitrile, 45% methanol, 100 mM ammonium acetate) to the sample. Pipet the mixture through the UMA-P tip up and down 30 times. Discard the mixture into an appropriate waste container.

5) Wash by pipetting and discarding 100 µl of the AMA solution through the UMA-P tip three times. Dispose the used AMA solution into an appropriate waste container.

6) Elute proteins with 30 µL of either 0.1% trifluoroacetic acid or 35% trifluoroethanol in 1% formic acid or 70% trifluoroethanol in 1% formic acid solution by pipetting up and down the UMA-P tip several times. 1% formic acid could be substituted with 0.1% trifluoroacetic acid.

**Supplementary Protocol 6**

Preparation of enzyme-embedded filter paper

This enzyme paper could be used for construction of different enzyme-embedded UMA units

1. Cut a 10x10 cm piece of filter paper.
2. Dissolve 150 µg of a lyophilised enzyme in 20 ml of deionized water.
3. Place the piece of paper into an appropriately-sized plastic dish and add the enzyme solution.
4. Incubate with a shaking at room temperature for 15 min.
5. Flip the piece of paper. Dispose of the enzyme solution.
6. Dissolve 150 µg of a lyophilised enzyme in 20 ml of deionized water.
7. Add this fresh enzyme solution to the paper.
8. Incubate with a shaking at room temperature for 15 min. Dispose of the enzyme solution.
9. Add 20 ml of deionized water.
10. Incubate with shaking for 2 min at room temperature. Dispose of the water.
11. Add 20 ml of ethanol. Incubate with shaking for 2 min. Dispose of the ethanol.
12. Let the filter paper dry out at room temperature for 24 hours. Store at 4^o^C.

**Supplementary Protocol 7**

Preparation of an enzyme-embedded UMA tip

1. An UMA tip is prepared by inserting two 2-mm plugs of the GB003 paper material (Whatman) into a 200-µl pipette tip.

2. ~2.5 µg of a lyophilised enzyme is dissolved in 180 µl of deionized water.

3. The UMA tip is prewashed with 100 µl of deionized water.

4. The enzyme solution is loaded into the UMA tip and slowly pushed out using a syringe with an appropriate tip adapter.

5. The UMA tip is washed with 100 µl of deionized water.

6. The UMA tip is washed with 100 µl of ethanol.

7. The UMA tip is left for 24 hours at room temperature to dry.

8. The UMA tip is stored at 4 ^o^C.

**Supplementary Protocol 8**

Basic UMA-T (UMA-Trypsin) Tip Embedded Trypsin Digestion Protocol

1) Cells are lysed in the basic SDS solubilization buffer (5% sodium dodecyl sulphate, 50 mM triethylammonium bicarbonate, pH ~8.0).

2) DNA is sheared by probe sonication. The sample is clarified by centrifugation for 5 min at ~13,000xg.

3) Protein disulphide bonds are reduced and cysteine residues alkylated (e.g. incubation with 20 mM dithiothreitol at 50 °C for 30 min and consequent incubation for 30 min at room temperature in the dark with 40 mM iodoacetamide).

4) The UMA-T tip can handle up to ~30 µg of protein load.

5) Add 6 volumes of the AMA solution (45% acetonitrile, 45% methanol, 100 mM ammonium acetate) to the sample. Mix by pipetting up and down several times. Add the mix into the UMA-T tip.

6) Spin the UMA-T tip in a bench-top centrifuge in a 2.0 ml sample tube with a column holder at ~2,500xg until all solution has passed through.

7) Wash by adding 150 µL of the AMA solution to the UMA-T tip. Spin at 5,000xg until all solution has passed through. The optional additional wash with the AMA solution could be performed.

8) Add 40 µl of 70 mM ammonium bicarbonate into the UMA-T tip. Place the UMA-T tip into a 1.5 ml sample collection tube. Push about half of the added solution through the UMA-T tip using a syringe with a tip adapter. Incubate the UMA-T tip/tube at 53 °C for 45 min.

9) Elute peptides with 40 µL of 70 mM ammonium bicarbonate.

10) Optional. Additional elution with 40 µL of 30% acetonitrile in 0.5% formic acid. Combine with the first elution.

11) Dry down the obtained eluate and re-suspend in 1% acetonitrile, 0.2% formic acid.

Important Note: Trifluoroethanol is classified as a hazardous substance and should be dealt with accordingly.
